# Research on improved convolutional wavelet neural network

**DOI:** 10.1038/s41598-021-97195-6

**Published:** 2021-09-09

**Authors:** Jingwei Liu, Peixuan Li, Xuehan Tang, Jiaxin Li, Jiaming Chen

**Affiliations:** 1 Information College, Capital University of Economics and Business, Beijing, 100070 China; 2 Information Department, Beijing University of Technology, Beijing, 100124 China

**Keywords:** Computational science, Computer science, Scientific data

## Abstract

Artificial neural networks (ANN) which include deep learning neural networks (DNN) have problems such as the local minimal problem of Back propagation neural network (BPNN), the unstable problem of Radial basis function neural network (RBFNN) and the limited maximum precision problem of Convolutional neural network (CNN). Performance (training speed, precision, etc.) of BPNN, RBFNN and CNN are expected to be improved. Main works are as follows: Firstly, based on existing BPNN and RBFNN, Wavelet neural network (WNN) is implemented in order to get better performance for further improving CNN. WNN adopts the network structure of BPNN in order to get faster training speed. WNN adopts the wavelet function as an activation function, whose form is similar to the radial basis function of RBFNN, in order to solve the local minimum problem. Secondly, WNN-based Convolutional wavelet neural network (CWNN) method is proposed, in which the fully connected layers (FCL) of CNN is replaced by WNN. Thirdly, comparative simulations based on MNIST and CIFAR-10 datasets among the discussed methods of BPNN, RBFNN, CNN and CWNN are implemented and analyzed. Fourthly, the wavelet-based Convolutional Neural Network (WCNN) is proposed, where the wavelet transformation is adopted as the activation function in Convolutional Pool Neural Network (CPNN) of CNN. Fifthly, simulations based on CWNN are implemented and analyzed on the MNIST dataset. Effects are as follows: Firstly, WNN can solve the problems of BPNN and RBFNN and have better performance. Secondly, the proposed CWNN can reduce the mean square error and the error rate of CNN, which means CWNN has better maximum precision than CNN. Thirdly, the proposed WCNN can reduce the mean square error and the error rate of CWNN, which means WCNN has better maximum precision than CWNN.

## Introduction

Artificial neural network (ANN)^1,2^ is a classic machine learning method. ANN is based on a collection of connected units or nodes called artificial neurons, which loosely models the neurons in a biological brain. Each connection, like the synapses in a biological brain, can transmit a signal from one artificial neuron to another. ANN can learn knowledge and use the learned knowledge to reason about results by the following two modes: Training mode (learning) and Forward calculation mode (reasoning). Convolutional neural network (CNN)^3,4^ is a method which based on feature extraction of convolution calculation.

The motivation of this study is as follows: Firstly, find a powerful simple neural network which has fast training speed like Back propagation neural network (BPNN)^5,6^ and Fully connected layers (FCL)^7–9^. Secondly, solve the local minimum problem according to Radial basis function neural network (RBFNN) method. Thirdly, improve the precision of CNN and verify the better performances of the proposed Convolutional wavelet neural network (CWNN) and Wavelet convolutional neural network (WCNN) based on the two well-known MNISIT and CIFAR-10 datasets.

Existing typical ANN and DNN methods are as follows: BPNN, RBFNN, Wavelet neural network^10,11^ (WNN), CNN, FCL, etc. Each of the above ANN (BPNN, RBFNN, WNN, and CNN are defined as XNN in this study) has advantages and disadvantages as follows: Firstly, the BPNN^12–14^ is a forward network. It is based on error back propagation^15^ and gradient descent algorithm. BPNN algorithm is widely used in many commercial applications. Advantages of BPNN are as follows: Firstly, BPNN has strong nonlinear mapping ability and high self-learning ability and adaptability; Secondly, BPNN can be wildly used because it can be well adapted to various samples; Problems and disadvantages of BPNN^16^ are as follows: Firstly, in the training process, the error of BPNN may drop into the local minimum; Secondly, in the training process, the convergence rate^17^ is slow. Secondly, RBFNN^18,19^ is a feedback network. The RBFNN hidden layer is composed of radial basis functions. Advantages of RBFNN are as follows: Firstly, RBFNN has no local minimum problem, which is the biggest problem of BPNN; Secondly, RBFNN has strong mapping ability from input to output and good classification ability. Problems and disadvantages of RBFNN are as follows: Firstly, it is very difficult to find the center of RBFNN hidden nodes; Secondly, it is difficult to determine the number of nodes in the hidden layer of RBFNN. Thirdly, CNN is a deep neural network^20,21^. A convolutional neural network consists of an input and an output layer, as well as multiple hidden layers. The hidden layers of a CNN typically consist of convolutional layers, activation function, pooling layers, fully connected layers and normalization layers. The advantage of CNN are as follows: CNN has more powerful learning ability for complex learning task. The disadvantage of CNN is that: when the learning object is too simple, the learning complexity of CNN is much bigger than the other ANNs. As a result, the learning speed of CNN may be slower than the other ANNs. Fourthly, FLC^22^ is one of the simplest neural networks, which has only two connected layers. The advantage of FLC is that: FLC has a very fast learning speed for simple learning cases than BPNN and RBFNN. The disadvantage of FCL is that: FLC cannot learn complex samples, even cannot complete some learning work which can be complete by BPNN and RBFNN.

Main contributions of this study are as follows: Firstly, the structure of BPNN, the radial basis function of RBFNN and the wavelet function are adopted to implement WNN. Secondly, based on WNN and CNN, CWNN is proposed to improve the performances. Thirdly, based on both MINIST and CIFAR-10 datasets, all the above discussed methods are compared.

The rest of this paper is organized as follows: “Results” section addresses the results obtained by three experiments on WNN and CWNN. "Methods" section details the methodology of WNN and CWNN. "Data" section introduces datasets and "Design of simulation" section introduces more details about all experiments. "Conclusions" section summaries all the simulation results and suggests some directions for further research.

## Results

Four experiments are implemented between BPNN, WNN, CWNN and WCNN in order to prove the improved effects. Firstly, "feasibility experiment" is designed to verify the feasibility (convergence) of WNN and prove that WNN can solve the problems of BPNN and RBFNN. Secondly, "performances experiment" is designed to verify the best performances (such as maximum precision, minimum error) of BPNN, RBFNN and WNN. Thirdly, "CWNN experiment" is designed in order to prove that the performances of the proposed CWNN is better than CNN. Fourthly, "WCNN experiment" is designed in order to prove that the performances of the proposed WCNN is better than CWNN and CNN.

### Definition 1

1 completed simulation process (1CSP) means a completed training process from beginning time 0 to the complete time (the time when the training error is less than the target error).

### Definition 2

1 simulation time (1CT) is only one training calculation. 1CSP contains many CTs. ${\Delta w}_{jk}^{(3)}$, $\Delta {w}_{ij}^{(2)}$, $\Delta {a}_{j}$, $\Delta {b}_{j}$ are calculated once in 1CT.

### Result of feasibility experiment

**Figure 1:**
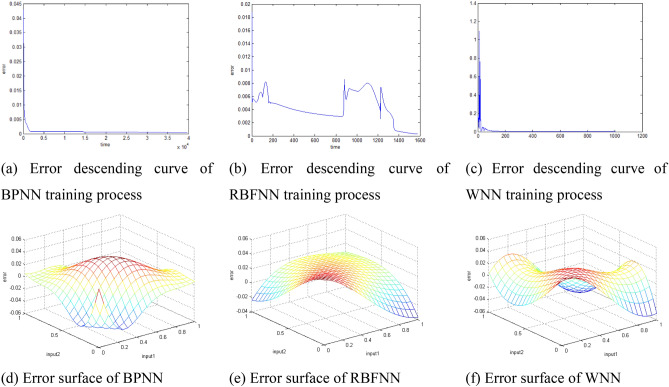
Error descending curve (time–error curve shows the descent process of error in 1CSP) was plotted in 2D figures. Error surface (all possible errors between 21 × 21 calculated outputs and target outputs) was plotted as 3D figures.

The dataset of "feasibility experiment" is generated by our designation which is specifically described in the section of "Data". Results of comparative simulations are discussed by two ways:

Firstly, error descending curves and error surfaces in 1CSP are plotted in Fig. 1.

All the simulations of BPNN, RBFNN and WNN are repeated for 10CSP. The condition to stop the simulation is that the target error in training process is less than a fixed value. Hence, the average CTs, the maximum error (between target value and calculated output) and the mean square error in each CSP can be calculated as follows:

The average CTs in each CSP of BPNN is 39,802. The maximum error is 0.050000. The mean square error is 0.000319. The error descending curve and error surface are drawn as Fig. 1a,d. The average CTs of RBFNN is 1580. The maximum error is 0.000314. The mean square error is 0.049570. The error descending curve and error surface are drawn as Fig. 1b,e. The average CTs of WNN is 1006. The maximum error is 0.000445. The mean square error is 0.049995. The error descending curve and error surface are drawn as Fig. 1c,f.

Secondly, statistical details of simulations in 10 CSPs are listed in Table 1, which are specifically discussed as follows:

**Table 1:** Simulation results of "Feasibility experiment".

Number of CSP	BPNN training times	BPNN mean square error	BPNN maximum error	RBFNN training times	RBFNN mean square error	RBFNN maximum error	WNN training times	WNN mean square error	WNN maximum error
1	1530	0.002328	0.099952	672	0.000966	0.099939	42	0.001329	0.096115
2	862	0.002457	0.099989	680	0.001164	0.093833	224	0.001596	0.099703
3	1791	0.002627	0.099962	319	0.001948	0.080631	173	0.001659	0.099784
4	972	0.002559	0.099991	985	0.001022	0.099876	954	0.001233	0.099798
5	1401	0.002637	0.099982	1417	0.001042	0.099998	713	0.001654	0.087587
6	1003	0.002255	0.099977	656	0.001518	0.099915	461	0.001911	0.099994
7	845	0.002162	0.099929	4464	0.001258	0.099445	222	0.001513	0.099781
8	2061	0.002355	0.099973	7401	0.000047	0.020000	111	0.001766	0.099980
9	1325	0.002577	0.099991	2330	0.001826	0.099944	478	0.001644	0.099883
10	1257	0.002481	0.099934	7374	0.001817	0.097768	53	0.001763	0.099677
Average	1305	0.002444	0.099968	2630 Slowest	0.001261	0.089135	343 Fastest	0.001607	0.098230
Success rate	100%			100%			100%		

10 CSPs of simulations of BPNN, RBFNN and WNN algorithms are compared to find out the differences of training times, mean square error and maximum error. Columns 2, 5, and 8 (XNN Training times) show how many CTs are required to complete the simulation in each CSP XNN. Columns 3, 6, and9 (XNN Mean square error) show the final mean square error after each training CSP. Columns 4, 7, and 10 (XNN maximum error) show the final maximum error after each training CSP. The maximum error is expected less than the target error. Therefore, if the training can be completed within 20,000 CTs, the maximum error is less than $\mathrm{err}\_\mathrm{goal}=0.1$. The results show that WNN can solve the problems of BPNN and RBFNN with better performance and make preparation for the improvement from CNN to CWNN.

According to the above Fig. 1 and Table 1, we can draw the following conclusions: Firstly, all of the BPNN, RBFNN and WNN algorithms are convergent. According to the columns 2, 5, and 8, all the values are less than the $max\_epoch=\mathrm{200,000}$, which means all the training process are convergent (all the training errors are lower than the target errors). It is proved that all the algorithms are feasible. Secondly, the average training times of WNN (CTs = 343) is the least, while the average training times of BPNN and RBFNN are 1305 and 2630. The average training times of RBFNN is the most. It is proved that WNN is the fastest algorithm, and RBFNN is the slowest one. Thirdly, the error descending curve of BPNN keeps decreasing, which causes problem of local minimum. BPNN also has problems such as slow convergence speed and local minimum problem^23^. However, the problem of local minimum are solved by RBFNN and WNN because of the structure of network and the active functions of RBFNN and WNN. The error curve of RBFNN in Fig. 1b not only reduces the time but also avoids the local minimum. While the error curve of WNN in Fig. 1c, significant changes (break the process of decreasing) only happen at the very beginning time of the training process.

### Result of hyperparameter optimization experiment

The "Hyperparameter optimization experiment" is designed and implemented in order to verify the best performances (max precision, min error) of BPNN, RBFNN and WNN, with the comparative results analyzed.

The simulation results of the above 3 algorithms are shown in Table 2, which are specifically discussed as follows.

**Table 2:** Simulation results of "Hyperparameter optimization experiment ".

Number of CSP	BPNN training times	BPNN mean square error	BPNN maximum error	RBFNN training times	RBFNN mean square error	RBFNN maximum error	WNN training times	WNN mean square error	WNN maximum error
1	20,000	0.000686	0.078208	20,000	0.007004	0.281643	1403	0.000052	0.019990
2	20,000	0.000441	0.052301	16,391	0.000078	0.019912	20,000	0.000261	0.032628
3	20,000	0.000559	0.056556	20,000	0.068413	0.762847	3740	0.000071	0.019316
4	20,000	0.000623	0.070849	7401	0.000047	0.020000	25e85	0.000065	0.019895
5	20,000	0.000687	0.082878	20,000	0.002712	0.136289	16,836	0.000030	0.019999
6	20,000	0.000610	0.071138	20,000	0.013012	0.401105	20,000	0.002192	0.109309
7	20,000	0.000572	0.068204	9546	0.000050	0.015604	1270	0.000048	0.019945
8	20,000	0.000601	0.070306	20,000	0.014854	0.338185	20,000	0.000183	0.039474
9	20,000	0.000617	0.070071	20,000	0.006495	0.211676	3943	0.000043	0.019992
10	20,000	0.000707	0.083616	5185	0.000073	0.019370	20,000	0.000562	0.063996
Mean	No case success	No case success	No case success	9631	0.000062	0.018722	4963 best	0.000052	0.019856
Success rate	0%			40%			60%best		

Columns 2, 5, and 8 (XNN Training times) show the numbers of CTs in each CSP. 20,000 means XNN cannot complete training within 200,000 CTs (i.e., the square error is not less than the target error with 20,000 training CTs). Columns 3, 6, and 9 (XNN Mean square error) show the final mean square error after each training CSP. Columns 4,7, and 10 (XNN Maximum error) show the final maximum error after each training CSP. The maximum error is expected less than the target error. Therefore, if the training can be completed within 20,000 CTs, the maximum error is less than $err\_goal$=0.02.

According to Table 2, we can draw the following conclusions: The success rate of WNN training is the highest (i.e., 60% of WNN training processes are completed), while only 40% of RBFNN training processes are completed, and 0% of BPNN training processes are completed. The precision of WNN is the highest because when the target precision ($errol\_goal$) is set from 0.1 to 0.02, the success rate of WNN is higher than RBFNN and BPNN. When the $errol\_goal$ is set from 0.02 to 0.005, only WNN can complete the training. The training speed of WNN is the fastest because the average training time of WNN (4936) is less than those of RBFNN (9631) and BPNN (20,000).

### Result of CWNN experiment

CWNN experiments are based on MNIST and CIFAR-10 datasets which are widely recognized and adopted in the comparative experiments.

#### CWNN is better than CNN on MNIST

Results on MNIST are recorded in Table 3. Error rate (effect of method) and Mean square error (MSE, effect of training) are two important indicators. According to the results on MNIST, main findings are as follows: Firstly, classification task can be completed by both CNN and CWNN. The change trend of MSE is declining during the training processes of both CNN and CWNN. Secondly, training accuracy of CWNN is better than CNN. Under the same training frequency, the MSE of CWNN (0.14146) is smaller than CNN (0.16431). Thirdly, classification ability of CWNN is better than CNN. Under the same training frequency, the error rate of CWNN (0.12915) is smaller than CNN (0.16434).

**Table 3:** Simulation results of CNN and CWNN on MNIST.

Numbers of SPs and statistical item	Total numbers of ACs	Error rate of CNN (accuracy)	MSE of CNN	Running times of CNN	Error rate of CWNN (accuracy)	MSE of CWNN	Running times of CWNN
1	6000	0.1584	0.1604	163.2180	0.1114	0.1368	217.7990
2	6000	0.1592	0.1593	164.2330	0.2453	0.2010	228.4130
3	6000	0.1578	0.1591	173.3970	0.1221	0.1250	218.7320
4	6000	0.1499	0.1532	173.3970	0.1088	0.1287	253.8580
5	6000	0.1701	0.1691	190.1890	0.1068	0.1330	244.1440
6	6000	0.1771	0.1759	185.5980	0.1425	0.1586	238.8930
7	6000	0.1599	0.1631	194.7600	0.1184	0.1386	534.7950
8	6000	0.1762	0.1747	191.8820	0.1173	0.1388	119.7400
9	6000	0.1541	0.1581	196.8820	0.1088	0.1267	282.4970
10	6000	0.1807	0.1702	237.1220	0.1101	0.1274	250.3990
Average	6000	0.16434 (0.83566)	0.16431	187.0700	0.12915(0.87085) Best	0.14146 Smaller	258.9270

Underlined values represent the results with the highest accuracy or the lowest mean square error.

#### CWNN is better than CNN on CIFAR-10

The results of CIFAR-10 are recorded in Table 4. Error rate and Mean square error are two important indicators. According to the results on CIFAR-10, main findings are as follows: Firstly, classification task can be completed by both CNN and CWNN. The change trend of MSE is declining during the training processes of both CNN and CWNN. Secondly, training accuracy of CWNN is better than CNN. Under the same training frequency, the MSE of CWNN (0.25715) is smaller than CNN (0.29845). Thirdly, classification ability of CWNN is better than CNN. Under the same training frequency, the error rate of CWNN (0.18522) is smaller than CNN (0.20510).

**Table 4:** Simulation results of CNN and CWNN on CIFAR-10.

Numbers of SPs and statistical item	Total numbers of ACs	Error Rate of CNN (accuracy)	MSE of CNN	Error rate of CWNN (accuracy)	MSE of CWNN
1	1000	0.1957	0.267	0.175239	0.242
2	1000	0.2611	0.4465	0.170323	0.2325
3	1000	0.1959	0.27	0.180921	0.257
4	1000	0.1888	0.263	0.177892	0.2455
5	1000	0.1985	0.2805	0.212793	0.2875
6	1000	0.2093	0.3135	0.192254	0.268
7	1000	0.2014	0.297	0.185923	0.267
8	1000	0.1884	0.26	0.172283	0.2335
9	1000	0.2048	0.2855	0.179464	0.25
10	1000	0.2068	0.3015	0.205191	0.2885
Average	1000	0.20510 (0.79489)	0.29845	0.18522 (0.81477) Smaller	0.25715 Smaller

Underlined values represent the results with the highest accuracy or the lowest mean square error.

### Result of WCNN experiment

WCNN experiments are based on MNIST datasets. WCNN is better than CWNN on MNIST. Results on MNIST are recorded in Table 5. According to the results on MNIST, main findings are as follows: Firstly, classification task can be completed by WCNN. The change trend of MSE is declining during the training processes. Secondly, training accuracy of WCNN is better than WCNN. Under the same training frequency, the MSE of WCNN (0.0.08850) is smaller than CWNN (0.14146). Thirdly, classification ability of WCNN is better than CWNN. Under the same training frequency, the error rate of WCNN (0.07723) is smaller than CWNN (0.18522).

**Table 5:** Simulation results of WCNN on MNIST.

Numbers of SPs and statistical item	Total numbers of ACs	Error rate of WCNN (accuracy)	MSE of WCNN	Running times of WCNN
1	6000	0.0478	0.0587	222.3220
2	6000	0.0566	0.0710	239.7420
3	6000	0.0977	0.1000	245.8560
4	6000	0.0501	0.0599	259.2540
5	6000	0.0680	0.0720	265.9660
6	6000	0.0473	0.0598	266.5450
7	6000	0.0366	0.0458	280.9490
8	6000	0.0397	0.0443	295.5480
9	6000	0.0701	0.0864	512.6480
10	6000	0.1954	0.1733	320.6640
11	6000	0.0576	0.0671	335.1010
12	6000	0.0654	0.0748	351.9140
13	6000	0.0483	0.0614	366.8210
14	6000	0.2870	0.3465	381.8980
15	6000	0.0763	0.0893	404.1410
16	6000	0.0452	0.0581	427.9340
17	6000	0.0670	0.0756	448.3010
18	6000	0.0573	0.0790	476.6720
19	6000	0.0483	0.0599	493.1710
20	6000	0.0860	0.0871	515.0280
Average	6000	0.07723(0.92277) Best	0.08850 Smaller	355.5238

Underlined values represent the results with the highest accuracy or the lowest mean square error.

## Methods

### Wavelet neural network (WNN)

The features, advantages and disadvantages of WNN^24,25^ are as follows: WNN has activation functions of multi-scaled analysis and scale translation in hidden layers. Advantages of WNN are as follows: Firstly, WNN has high precision and high resolution. Secondly, wavelet transform method is good at analyzing local information of signals^26,27^ which is also the feature of WNN. Thirdly, wavelet transform can do an excellent job such as function approximation^28,29^ and pattern classification^30^. It has been proved that the wavelet neural network is an excellent approximator for fitting single variable function^31^. Problems and disadvantages of WNN are as follows: WNN cannot complete complex learning task because of structural limitation, etc. Similar problems also exist in BPNN, RBFNN and FCL.

**Figure 2:**
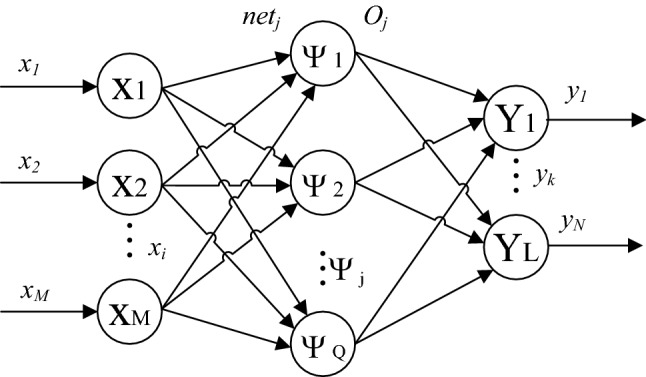
Structure of WNN. From left to right, there is an input layer, a hidden layer, and an output layer. $net$ represents the inputs of hidden layer and $O$ represents for the outputs of hidden layer.$\Psi$ represents the wavelet function (activation function).

WNN is designed as follows: Firstly, structure of BPNN is adopted as the basic structure of WNN; Secondly, the form of activation function in hidden layers of RBFNN is adopted; Thirdly, the wavelet transform function is adopted as the activation function. The structure of WNN is shown in Fig. 2.

The output neurons of hidden layer ${O}_{j}^{-2}\left(t\right)$ can be expressed as Eq. (1):

1 $${O}_{j}^{-2}\left(t\right)={\Psi }_{a,b}\left(\frac{{net}_{j}^{-2}(t)-{b}_{j}(t)}{{a}_{j}(t)}\right)$$

In Eq. (1), ${{net}_{j}^{-2}\left(t\right)\mathrm{ is the input of neurons j in hidden layer}. \Psi }_{a,b}(t)$ is the wavelet function (activation function). Parameters ${a}_{j}\left(t\right)$ and ${b}_{j}\left(t\right)$ are the scaling parameters of the wavelet function. $t$ is the number of training time.

The number of nodes in hidden layer can be calculated according to the linear correlation theory: Redundant (repetitive or useless) nodes can be found and deleted by the comparison of parameters ${\Psi }_{a,b}\left(t\right)$ in each node in hidden layer. The wavelet function (activation function) can be selected according to the frame theory. The closer the frame is to the boundary, the better the stability of the wavelet function, but when the frame is closer to the boundary, the problem of data redundancy will occur.

The wavelet function that satisfies the framework conditions is selected in Eq. (2):

2 $${\Psi }_{a,b}\left(t\right)=cos(1.75t)\cdot {e}^{-\frac{{t}^{2}}{2}}$$

The loss function that we select is mean square error(MSE). $E$ is the mean square error (MSE) of all samples, which can be formulated as Eq. (3). The reasons we use the mean square error (MSE) are as follows: Firstly, the outputs of WNN have negative numbers. Secondly, the cross-entropy loss function includes the logarithm function, which requires non-negative inputs.

3 $$E=\frac{1}{2}\sum_{n=1}^{N}{({\widehat{y}}_{n}-{y}_{n})}^{2}$$

In WNN, the back propagation of input errors (${\delta }_{k}^{-3}$ in the output layer, ${\delta }_{j}^{-2}$ in the hidden layer and ${\delta }_{i}^{-1}$ in the input layer) can be calculated as Eq. (4) to Eq. (6).

4 $${\updelta }_{i}^{-1}=\frac{\partial E}{\partial {net}_{i}^{-1}}=\frac{1}{N}\sum_{n=1}^{N}({\widehat{y}}_{n}-{y}_{n})(1-{net}_{i}^{-1}){net}_{i}^{-1} ,\mathrm{ i}=\mathrm{1,2},\dots ,{size}^{-1}$$

5 $${\updelta }_{j}^{-2}=\frac{\partial E}{\partial {net}_{j}^{-2}}=\frac{\partial E}{\partial {net}_{k}^{-1}}\cdot \frac{\partial {net}_{k}^{-1}}{\partial {O}_{j}^{-2}}\cdot \frac{\partial {O}_{j}^{-2}}{\partial {net}_{j}^{-2}}=\frac{1}{{b}_{j}^{-2}}\sum_{k=1}^{{size}^{-1}}{\updelta }_{k}^{-1}\cdot {\Psi }^{\mathrm{^{\prime}}\left(\frac{{net}_{j}^{-1}-{a}_{j}^{-2}}{{b}_{j}^{-2}}\right)}\cdot {w}_{ij}^{-1}, j=1,\dots ,{size}^{-2}$$

6 $${\updelta }_{k}^{-3}=\frac{\partial E}{\partial {net}_{k}^{-3}}=\frac{\partial E}{\partial {O}_{k}^{-3}}=\frac{\partial E}{\partial {net}_{k}^{-2}}\cdot \frac{\partial {net}_{j}^{-2}}{\partial {O}_{k}^{-3}}=\sum_{j=1}^{{size}^{-2}}{\updelta }_{j}^{-2}\cdot {w}_{kj}^{-2},\mathrm{ k}=\mathrm{1,2},\dots ,{size}^{-3}$$

Gradient descent method is adopted to adjust weights and bias of the neural network. Parameters such as ${\Delta w}_{ij}^{-2}$, $\Delta {a}_{j}^{-2}$, $\Delta {b}_{j}^{-2}$, $\Delta {w}_{kj}^{-1}$,$\Delta {b}_{k }^{-1}$ are adjusted in each training process. $i$*,* $j$ and $k$ are the numbers of neuron in each layer. ${\Delta w}_{ij}^{-2}$ represents the changed values of weight between the neurons in the input layer and the hidden layer. $\Delta {a}_{j}^{-2}$ and $\Delta {b}_{j}^{-2}$ represents the changed values of bias between the input layer and the hidden layer. $\Delta {w}_{kj}^{-1}$ represents the changed values of weight between the neurons in the hidden layer and the output layer.$\Delta {b}_{k }^{-1}$ represents the changed values of bias between the hidden layer and the output layer. ${net}_{j}^{-2}$ represents the input of hidden layer. At the training time $t$, the above parameters can be expressed as Eq. (7) to Eq. (11):

7 $${\Delta w}_{ij}^{-2}=\frac{\partial E}{{\partial w}_{ij}^{-2}}=\frac{\partial E}{\partial {net}_{j}^{-2}}\times \frac{\partial {net}_{j}^{-2}}{{\partial w}_{ij}^{-2}}={\updelta }_{j}^{-2}\cdot {O}_{i}^{-3}$$

8 $$\Delta {a}_{j}^{-2}=\frac{\partial E}{\partial {a}_{j}^{-2}}=\frac{\partial E}{\partial {net}_{j}^{-2}}\times \frac{\partial {net}_{j}^{-2}}{\partial {a}_{j}^{-2}}=\frac{1}{{size}^{-2}}\sum_{j=1}^{{size}^{-2}}-{\delta }_{j}^{2}\cdot \frac{1}{{b}_{j}^{-2}}$$

9 $$\Delta {b}_{j}^{-2}=\frac{\partial E}{\partial {b}_{j}^{-2}}=\frac{\partial E}{\partial {net}_{j}^{-2}}\times \frac{\partial {net}_{j}^{-2}}{\partial {b}_{j}^{-2}}=-\frac{1}{{size}^{-2}}\sum_{j=1}^{{size}^{-2}}\frac{1}{{{(b}_{j}^{-2})}^{2}}\cdot {\delta }_{j}^{-2}\cdot ({net}_{j}^{-1}-{a}_{j}^{-2})$$

10 $$\Delta {w}_{kj}^{-1}=\frac{\partial E}{{\partial w}_{kj}^{-1}}=\frac{\partial E}{\partial {net}_{k}^{-1}}\times \frac{\partial {net}_{k}^{-1}}{\partial {w}_{kj}^{-1}}={\updelta }_{k}^{-1}\cdot {O}_{j}^{-2}$$

11 $$\Delta {b}_{k }^{-1}=\frac{\partial E}{\partial {b}_{k }^{-1}}=\frac{\partial E}{\partial {net}_{k}^{-1}}\times \frac{\partial {net}_{j}^{-1}}{\partial {b}_{k}^{-1}}={\updelta }_{k}^{-1}$$

The adjusted results of the above weights and bias are expressed as Eq. (12) to Eq. (16), where $\alpha \_WNN$ is the inertia coefficient of WNN, $\eta \_WNN$ is the learning rate of WNN.

12 $${w}_{ij}^{-2}\left(t+1\right)={w}_{ij}^{-2}\left(t\right)-{\Delta w}_{ij}^{-2}\times \eta \_\mathrm{W}NN+{w}_{ij}^{-2}\left(t\right)\times \alpha \_\mathrm{W}NN$$

13 $${a}_{j}^{-2}\left(t+1\right)={a}_{j}^{-2}\left(t\right)-{\Delta a}_{j}^{-2}\times \eta \_\mathrm{W}NN+{a}_{j}^{-2}\left(t\right)\times \alpha \_\mathrm{W}NN$$

14 $${b}_{j}^{-2}\left(t+1\right)={b}_{j}^{-2}\left(t\right)-\Delta {b}_{j}^{-2}\times \eta \_\mathrm{W}NN+{b}_{j}^{-2}\left(t\right)\times \alpha \_\mathrm{W}NN$$

15 $${w}_{kj}^{-1}\left(t+1\right)={w}_{kj}^{-1}\left(t\right)-\Delta {w}_{kj}^{-1}\times \eta \_\mathrm{W}NN$$

16 $${b}_{k }^{-1}\left(t+1\right)={b}_{k }^{-1}\left(t\right)-\Delta {b}_{k }^{-1}\times \eta \_\mathrm{W}NN$$

According to the above descriptions, pseudocode of WNN method for simulations is shown in Algorithm 1.
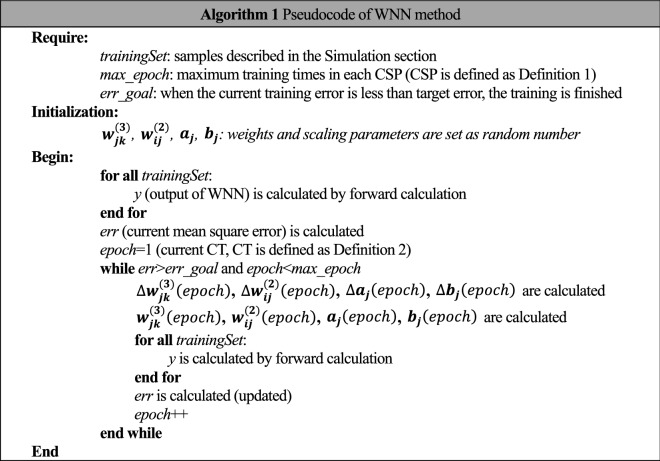


### Convolutional wavelet neural network (CWNN)

**Figure 3:**
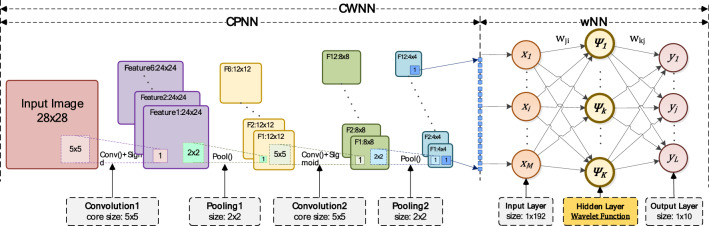
Structure of CWNN. CWNN is composed of CNN and WNN. CNN consists the convolution layer and the pooling layer. The fully connected neural network (FCNN) of CNN is replaced by WNN.

Based on CNN, the improvement of CWNN is that: the fully connected neural network (FCNN) of CNN is replaced by WNN. The structure of CWNN has two parts that including convolutional and pooling layer of neural network (CPNN) and WNN. In the hidden layer of the WNN, the activation functions are wavelet scale transformation functions. The structure of CWNN is drawn as Fig. 3.

Training algorithm of CWNN is similar to CNN, while the difference is that FCNN in CWNN is replaced by WNN in CWNN. The pseudocode of CWNN is listed in Algorithm 2.
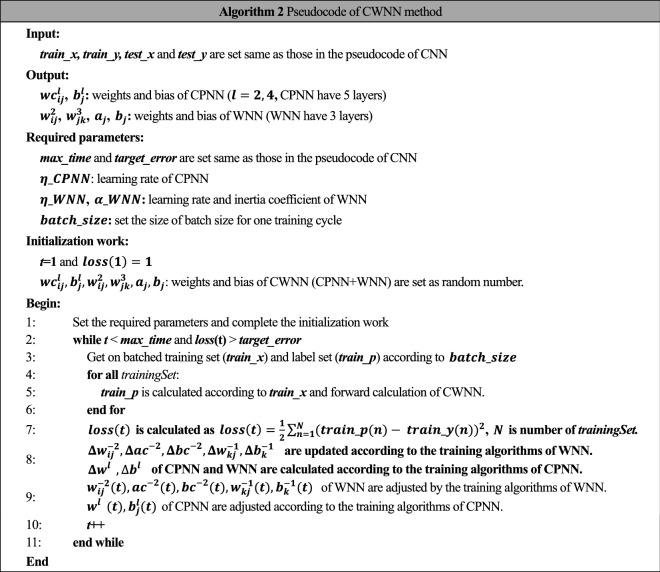


### Wavelet convolutional neural network (WCNN)

The improvement of the proposed WCNN is that: the activation function of the convolutional layer in CNN is replaced by the $\Psi ()$. The activation function of CNN is sigmoid function, and the $\Psi ()$ of WCNN is wavelet scale transformation function.

**Figure 4:**
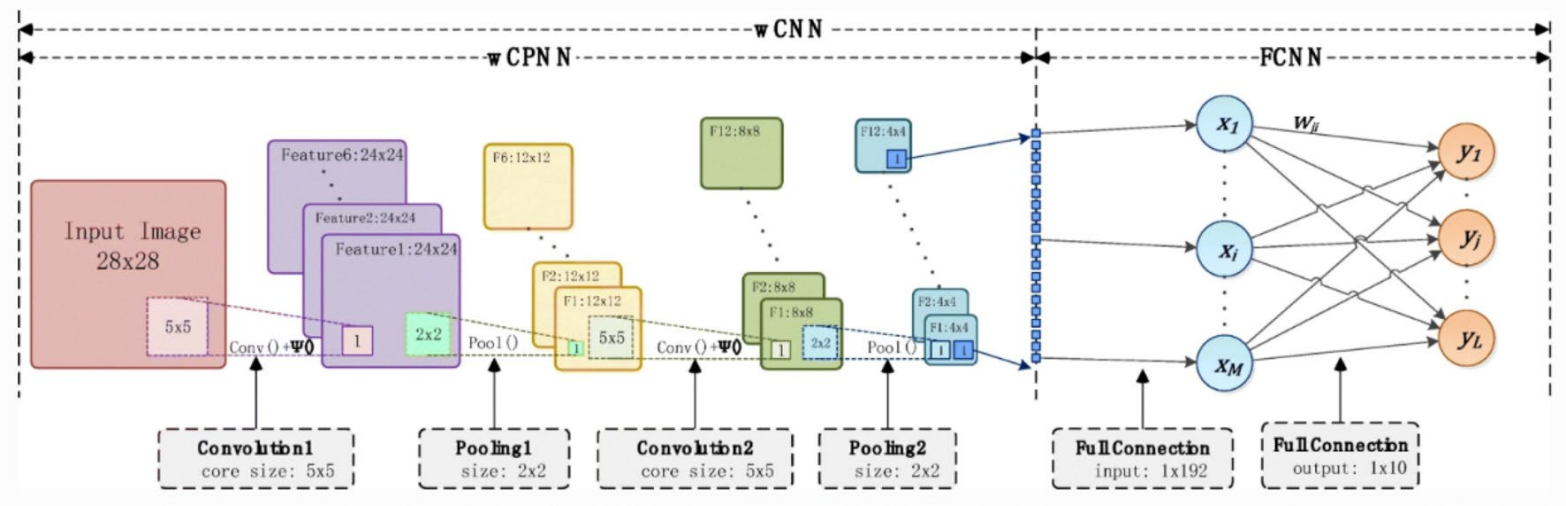
Structure of WCNN. WCNN is composed of WCPNN and FCNN. The first part of WCPNN is Wavelet Convolutional Pooling Neural Network (WCNN) and the activation function of WCNN is wavelet scale transformation function.

The structure of proposed WCNN is that: The first part of WCPNN is Wavelet Convolutional Pooling Neural Network (WCPNN), and the second part is Fully Connected Neural Network (FCNN). The structure of WCNN is shown in Fig. 4.

The training process of WCNN is similar to CNN, while the activation function of WCNN is different from CNN. The pseudocode of WCNN is listed in Algorithm 3.
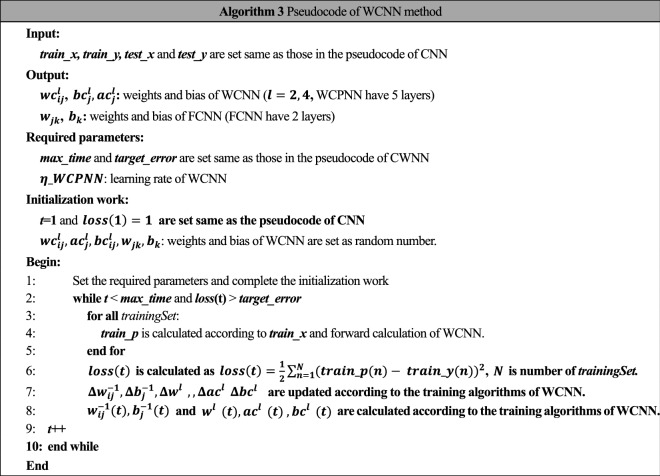


## Data

### Datasets generation

The data of the first two experiments ("feasibility experiment" and "Hyperparameter optimization experiment ") can be generated in the following steps.

The first step is to generate the training set: The training set has two features: x and y. The label of the training set is z = f (x, y). The relationship between the two-dimensional features and the one-dimensional label can be expressed in Eq. (17):

17 $$\mathrm{Z}\left(\mathrm{x},\mathrm{y}\right)=\mathrm{sin}\left(90\cdot x\right)+\mathrm{cos}\left(90\cdot y\right)$$

According to Eq. (17), features of dataset ($F$) can be generated as Eq. (18). There are 3 different values in $F$, hence there are 3 × 3 = 9 groups of features. Label of dataset ($L$) can be calculated as Eq. (19):

18 $$F=\left[\begin{array}{c}\begin{array}{ccc}\begin{array}{ccc}0& 0& 0\end{array}& \begin{array}{ccc}0.5& 0.5& 0.5\end{array}& \begin{array}{ccc}1& 1& 1\end{array}\end{array}\\ \begin{array}{ccc}\begin{array}{ccc}0& 0.5& 1\end{array}& \begin{array}{ccc}0& 0.5& 1\end{array}& \begin{array}{ccc}0& 0.5& 1\end{array}\end{array}\end{array}\right]$$

19 $$L=\left[\begin{array}{ccc}\begin{array}{ccc}0& 0.3& 0.5\end{array}& \begin{array}{ccc}0.35& 0.71& 0.85\end{array}& \begin{array}{ccc}0& 0.5& 1\end{array}\end{array}\right]$$

The second step is to generate the test set: The features ${F}_{t}$ are designed with 21 different values, hence there are 21 × 21 = 441 groups of features. According to Eq. (18), ${F}_{t}$ can be designed as a matrix with 2 rows and 441 columns. Each column of ${F}_{t}$ represent one group of features. The test dataset can be generated by the pseudocode as Algorithm 3.
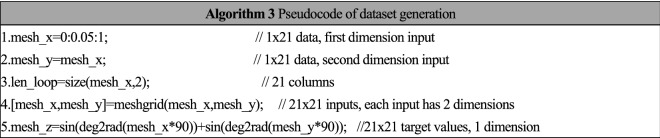


### Datasets of MNISIT and CIFAR-10

**Figure 5:**
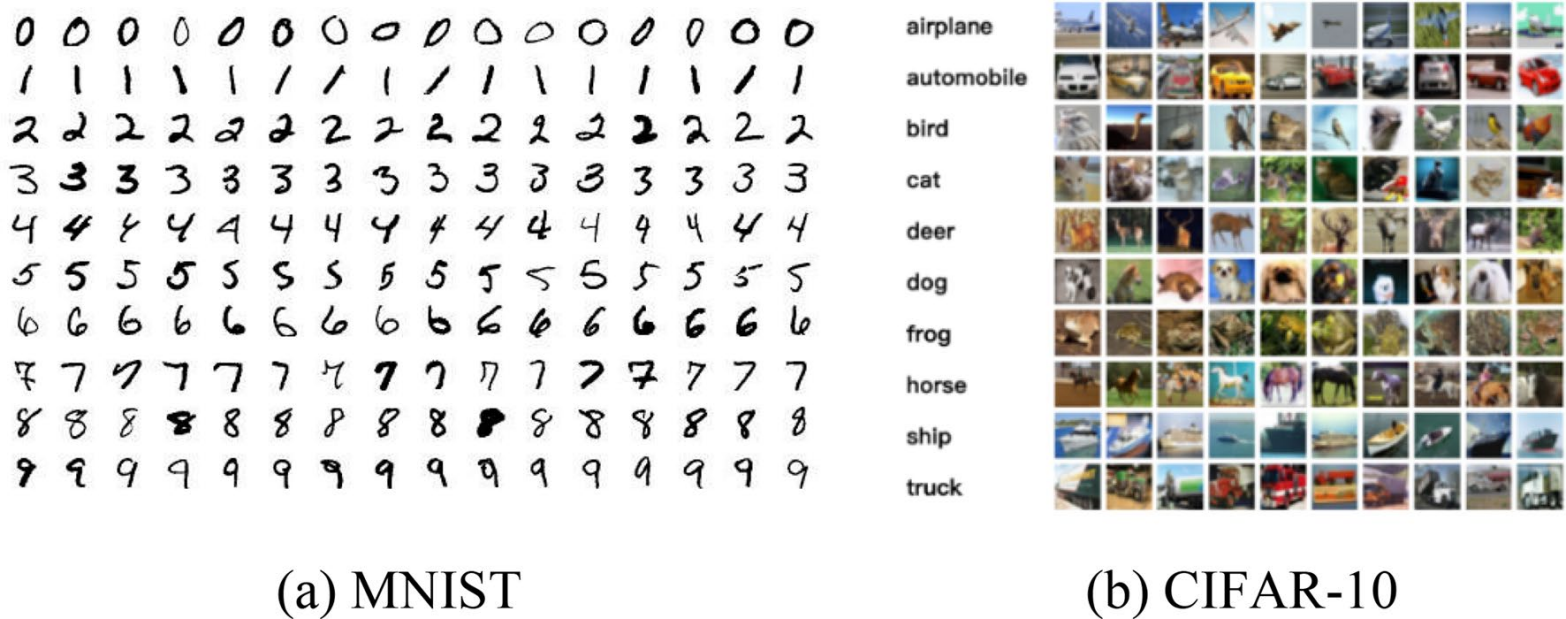
Datasets. (**a**) MNIST is composed of many handwritten from different people. Training set contains of 250 digits and test set consists of the same proportion of digits data. (**b**) CIFAR-10 is widely used in lots of image classification research. There are 50,000 32 × 32 images in the training set and 10,000 32 × 32 images in the test set.

MNISIT and CIFAR-10 are widely recognized and adopted in the comparative experiments. MNIST is well known from the National Institute of Standards and Technology. In MNIST, the training set consists of 250 digits handwritten from different people, and test set consists of the same proportion of digits data. CIFAR-10 is widely used in lots of image classification research. In CIFAR-10, there are 50,000 32 × 32 images in the training set and 10,000 32 × 32 images in the test set. To meet the need of comparison, the size of samples in CIFAR-10 is reshaped to 28 × 28. All samples are divided into 10 classes, with 6000 samples in each class. Images in the two datasets are shown in Fig. 5.

## Design of simulation

### Architecture of WNN

"Feasibility experiment" is designed as follows: Common parameters for all the algorithms are set according to the same rules as follows: Firstly, the initial parameters such as ${w}_{jk}$, ${w}_{ij}$ are set with the same randomization rules. Secondly, the target precision is set as a very low value (i.e., the target error is set as a very big value), so all the above algorithms are very easy to converge. Thirdly, the training time is set as a large value, so all the above algorithms have enough training time to complete the training. The initialization work for specific parameters is as follows: Firstly, maximum training time limitation is set as a very large number ($max\_epoch=\mathrm{20,000}$) to ensure that BPNN, RBFNN, WNN have enough time to complete training. Secondly, target error is set as a very large number ($err\_goal=0.1$) to make the training easy to complete. Thirdly, training processes of each algorithm are repeated 10 times (10 CSPs, CSP is defined in definition 2, i.e., $case\_repeat=10$) to observe statistical characteristics. Fourthly, the learning efficiency parameter is set as $lr=0.2$. Fifthly, the inertia coefficient parameter is set as $la=0.3$. Many values for $lr$ and $la$ were tested, where $0.2$ and $0.3$ were optimal for $lr$ and $la$, respectively. The termination condition is that: Firstly, when the current training error is less than the target error, the training process is completed. Secondly, when the current training time exceeds the maximum training time limit, the training is stopped.

"Hyperparameter optimization experiment" is designed as follows: Parameters for this simulation are set as follows: Firstly, the initial parameters such as ${w}_{jk}$*,*${w}_{ij}$ were set with the same randomization rules. Secondly, the target precision was set very high to find the highest precision of BPNN, RBFNN, and WNN. Thirdly, the training time was set as a large value, so all the above algorithms have enough training time to complete the training. The initialization work for specific parameters is as follows: Firstly, maximum training time limitation was set as a very large number (*max_epoch* = 20,000). Secondly, maximum error was set as a very small number (err_goal = 0.02). Thirdly, simulations of BPNN, RBFNN, and WNN were repeated for 10 CSPs (case_repeat = 10). Fourthly, the learning efficiency parameter was set as lr = 0.2. Fifthly, the inertia coefficient parameter was set as la = 0.3.More values of lr and la were tested in different simulations, and the values of lr = 0.2 and la = 0.3 listed above are the best. The termination condition is that: Firstly, when the current training error is less than the target error, the training process is completed. Secondly, when the current training time exceeds the maximum training time limit, the training is stopped.

### Architecture of CWNN

Simulation of CWNN is designed as follows: The network structures and parameters of CNN and CWNN simulation are listed in Table 6. Different values of the learning rate $\eta$ and coefficient of inertia $\alpha$ are tested in repetitive simulations, and the values of $\eta$ and $\alpha$ listed in Table 6 are the best ones.

**Table 6:** Configurations of CNN, CWNN experiments.

No.	Parameter type	Parameter name	CNN	CWNN
1	1st to 5th layers	First type of NN	CPNN	CPNN
2	2nd, 4th layers	Activation function of convolutional layer	Sigmoid	Sigmoid
3	1st layer	Dimension of the 1st layer	$28\times 28$	$28\times 28$
4	2nd layer	Dimension of 1st convolutional layer	$28\times 28$	$28\times 28$
5	3rd layer	Dimension of 1st pooling layer	$24\times 24$	$24\times 24$
6	2nd, 3rd layers	Number of features	$6$	$6$
7	4th layer	Dimension of 2nd convolutional layer	$12\times 12$	$12\times 12$
8	5th layer	Dimension of 2nd 1 pooling layer	$8\times 8$	$8\times 8$
9	4th, 5th layers	Number of features	$12$	$12$
10	− 1st, − 2nd, − 3rd layers	Second type of NN	FCNN	WNN
11	− 3rd layer	Dimension of -3rd input layer	192	192
12	− 2nd layer	Dimension of -2nd hidden layer	None	$50$
13	− 2nd layer	Activation function of hidden layer	None	Wavelet
14	− 1st layer	Dimension of − 1st output layer	10	10
15	− 1st layer	Activation function of output layer	Sigmoid	Sigmoid
16	Hyperparameters	Learning rate $\eta$	0.1	0.1
17	Hyperparameters	Coefficient of inertia $\mathrm{\alpha }$	None	0.2
18	Hyperparameters	$max\_SPs$	10	10
19	Hyperparameters	$max\_ACs$	6000	6000
20	Hyperparameters	$target\_err$	0.0000001	0.0000001
21	Hyperparameters	$BatchSize$	10	10

### Architecture of WCNN

Simulation of WCNN is designed as follows: The network structures and parameters of WCNN simulation are listed in Table 7. Different values of the learning rate $\eta$ and other parameters are tested in repetitive simulations, and the values of $\eta$ in Table 7 are the best ones.

**Table 7:** Configurations of WCNN experiments.

No.	Parameter type	Parameter name	WCNN
1	1st to 5th layers	First type of NN	WCPNN
2	2nd, 4th layers	Activation function of convolutional layer	Wavelet
3	1st layer	Dimension of the 1st layer	$28\times 28$
4	2nd layer	Dimension of 1st convolutional layer	$28\times 28$
5	3rd layer	Dimension of 1st pooling layer	$24\times 24$
6	2nd, 3rd layers	Number of features	$6$
7	4th layer	Dimension of 2nd convolutional layer	$12\times 12$
8	5th layer	Dimension of 2nd 1 pooling layer	$8\times 8$
9	4th,5 th layers	Number of features	$12$
10	− 1st, − 2nd, − 3rd layers	Second type of NN	FCNN
11	− 3rd layer	Dimension of -3rd input layer	192
12	− 2nd layer	Dimension of -2nd hidden layer	None
13	− 2nd layer	Activation function of hidden layer	None
14	− 1st layer	Dimension of -1st output layer	10
15	− 1st layer	Activation function of output layer	Sigmoid
16	Hyperparameters	Learning rate $\eta$	0.1
17	Hyperparameters	Coefficient of inertia $\mathrm{\alpha }$	None
18	Hyperparameters	$max\_SPs$	10
19	Hyperparameters	$max\_ACs$	6000
20	Hyperparameters	$target\_err$	0.0000001
21	Hyperparameters	$BatchSize$	10

## Conclusions

In this paper, WNN, CNN are implemented and CWNN, WCNN are proposed, all of them are simulated and compared. Conclusions are as follows:

Firstly, both the structure of BPNN, the form of activation function in hidden layers of RBFNN and the wavelet transform functions are adopted to the design of WNN. The comparative results of BPNN,RBFNN and WNN are shown in Table 8.

**Table 8:** Comparative results of BPNN, RBFNN and WNN on generation dataset.

Statistics	BPNN	RBFNN	WNN
Maximum MSE	0.000707	0.068413	0.002192
Minimum MSE	0.00061	0.00005	0.00003
Mean MSE	0.00061	0.011274	0.000351
Maximum error rate	0.083616	0.762847	0.109309
Minimum error rate	0.052301	0.015604	0.019316
Mean error rate	0.070413	0.220663	0.036454
Mean training times	No case success	9631	4963

According to Table 8, the following conclusions can be drawn: the mean MSE and mean error rate of WNN are lowest, and the training speed of WNN is the fastest, and the WNN method has no local minimum issue.

Secondly, CWNN is proposed. The fully connected neural network (FCNN) of CNN is replaced by WNN. WCNN is proposed. The activation function of the convolutional layer in CNN is replaced by the wavelet scale transformation function. The comparative simulations between CNN,CWNN and WCNN are shown in Table 9.

**Table 9:** Comparative results of CNN, CWNN and WCNN on MNIST dataset.

Statistics	CNN	CWNN	WCNN
Maximum MSE	0.1759	0.201	0.3456
Minimum MSE	0.1532	0.125	0.03456
Mean MSE	0.1643	0.1414	0.0885
Maximum error rate	0.1807	0.2453	0.287
Minimum error rate	0.1499	0.1068	0.0366
Mean error rate	0.1643	0.1291	0.0772

According to Table 9, the following conclusions can be drawn: All of CNN, CWNN and CWNN can complete the task of classification on MNIST.

Fifthly, training accuracy of CWNN is higher than CNN and classification ability of CWNN is better than CNN. Fifthly, training accuracy of WCNN is higher than CWNN and classification ability of WCNN methods are better than CWNN.

There are still some limitations of our methods although we've made some improvements based on CNN. Firstly, limitations of convolution layer. Back propagation algorithm is not a very efficient learning method, because these algorithms need the support of large-scale data sets. In back propagation algorithm, the parameters near the input layer will be adjusted very slowly when the layers are too deep. Secondly, limitations of pooling layer. A lot of valuable information such as information between the local and the whole will be lost in the pooling layer. Finally, the features extracted from each convolution layer cannot be explained, because neural network is a black box model which is difficult to be explained.

For the further research: Firstly, try to improve the learning ability and learning speed of CNN, WCNN and CWNN by changing the network structure. Secondly, use WCNN and CWNN as neurons to build a more lager and powerful neural network. Thirdly, design more experiments to prove the feasibility and verify the performances of the improved methods and of the work above.
